# Optoelectronic Plethysmography has Improved our Knowledge of Respiratory Physiology and Pathophysiology

**DOI:** 10.3390/s8127951

**Published:** 2008-12-05

**Authors:** Isabella Romagnoli, Barbara Lanini, Barbara Binazzi, Roberto Bianchi, Claudia Coli, Loredana Stendardi, Francesco Gigliotti, Giorgio Scano

**Affiliations:** Don C. Gnocchi Foundation ONLUS, via Imprunetana 124, 50023 Pozzolatico, Florence, Italy; E-Mails: iromagnoli@dongnocchi.it (I. R.); blanini@dongnocchi.it (B. L.); babybin@libero.it (B. B.); coccio7@tin.it (R. B.); c.claud@libero.it (C. C.); lstendardi@libero.it (L. S.); fgigliotti@dongnocchi.it (F. G.)

**Keywords:** Chest wall kinematics, lung volumes, rib cage distortion, respiratory muscles, expiratory flow limitation, breathing pattern, dyspnea

## Abstract

It is well known that the methods actually used to track thoraco-abdominal volume displacement have several limitations. This review evaluates the clinical usefulness of measuring chest wall kinematics by optoelectronic plethysmography [OEP]. OEP provides direct measurements (both absolute and its variations) of the volume of the chest wall and its compartments, according to the model of Ward and Macklem, without requiring calibration or subject cooperation. The system is non invasive and does not require a mouthpiece or nose-clip which may modify the pattern of breathing, making the subject aware of his breathing. Also, the precise assessment of compartmental changes in chest wall volumes, combined with pressure measurements, provides a detailed description of the action and control of the different respiratory muscle groups and assessment of chest wall dynamics in a number of physiological and clinical experimental conditions.

## 1. Introduction

The precise assessment of changes in thoraco-abdominal volumes, combined with pressure measurements, allows a detailed description of the action and control of the different respiratory muscle groups. That is the reason why the accurate computation of thoraco-abdominal volume changes is needed. It is well known that methods actually in use for the computation of thoraco-abdominal volume displacement are affected by several limitations. The most used devices able to compute dynamic changes of the thoraco-abdominal wall are magnetometers and inductance plethysmography (Respitrace^®^). Both these systems are based on the assumption that the thoraco-abdominal wall has only two degrees of freedom but it is well known that changes in both antero-posterior diameter and changes in cross-sectional area of thoracic and abdominal compartments are not linearly related to their respective volumes. Furthermore both devices are strongly influenced by artifacts due to the subject posture [1, 2] that limit their utilization in dynamic conditions (e.g. exercise).

An ideal system able to measure movements and volumes of the respiratory system should have as many as possible of the following characteristics:

1. Accurate computation of volume changes without using a mouthpiece that may alter the normal breathing pattern [3].
2. Need of a simple, stable and repeatable calibration.
3. Possibility of use in non-collaborating subjects (during sleep, or in unconscious patients).
4. Permitting the analysis in different postures.
5. Permitting the analysis under dynamic conditions such as walking or cycling.
6. Allowing high frequency response in order to accurately describe rapid phenomena (i.e. electric or magnetic stimulation of phrenic nerves).
7. Allowing the analysis of movements and volume changing of the different compartments of the chest wall: the upper thorax, lower thorax, and abdomen).
8. Allowing the analysis of movements and volume changing of the two half (left and right) of the chest wall.
9. Being non-invasive and safe for the patient.

An OEP device able to track the three-dimensional co-ordinates of a number of reflecting markers placed non-invasively on the skin of the subject satisfies many of these characteristics. The simultaneous acquisition of kinematic signals with pleural and gastric pressures during a relaxation manoeuvre allows the representation of pressure-volume plots describing the mechanical characteristics of each compartment. The OEP System was developed in 80's by the Bioengineering Department of the University of Milano in order to overcome as many of the previously mentioned limitations as possible [4-7].

## 2. Methods

**Figure 1.**
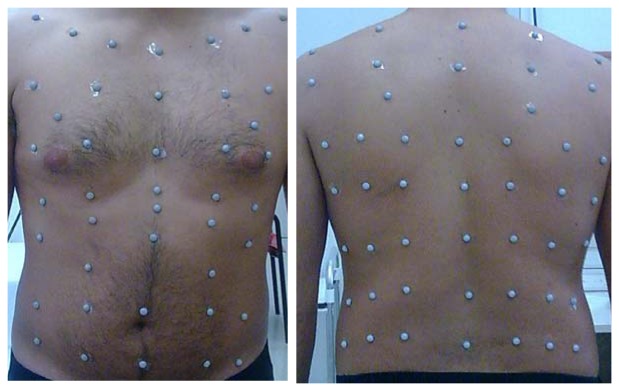
89 marker model for respiratory acquisition. 42 markers are placed in front and 47 on the back of the subject.

OEP system is an optoelectronic device able to track the three-dimensional co-ordinates of a number of reflecting markers placed non-invasively on the skin of the subject [4-7]. A variable number of markers (89 in the model used for respiratory acquisition in seated position) is placed on the thoraco-abdominal surface; each marker is a half plastic sphere coated with a reflective paper. Two TV cameras are needed to reconstruct the X-Y-Z co-ordinates of each marker, so for the seated position two pairs of cameras are required. Each set of cameras is aligned vertically: one near the ceiling and the other near the floor. Each camera is equipped with an infra-red ring flash. This source of illumination, which is not visible, is not disturbing and lets the system also operate in the dark (a condition required during sleep studies). The infra-red beam, emitted by the flashes, is reflected by each marker and acquired by the cameras with a maximal sampling rate of 100 Hz. The signal is then processed by a PC board able to combine the signal coming from the two cameras and to return, frame by frame, the three-dimensional co-ordinates of each marker. The process is simultaneously carried out for the two pairs of TV cameras needed for the seated respiratory model. Acquired data need a further operation called ‘tracking’ that is necessary to exclude possible phantom reflections and/or to reconstruct possible lost markers (this could happens sometimes during very fast manoeuvres such exercise); at this time the obtained files contain the X-Y-Z co-ordinates of each marker during the recorded manoeuvre, then data are stored on the PC hard disk. The spatial accuracy for each marker's position is about 0.2 mm [4]. Volumes for each compartment is calculated by constructing a triangulation over the surface obtained volume from the X-Y-Z co-ordinates of the markers and then using Gauss's theorem to convert the volume integral to an integral over this surface [5]. The number and the position of used markers depends on the thoraco-abdominal model chosen. As proposed by Ward & Macklem [8] we use a three compartment chest wall model: the upper rib cage [RC,p], lower Rib cage [RC,a] and abdomen [AB]. Due to the fact that the upper portion of the rib cage is exposed to pleural pressure whereas the lower portion is affected by abdominal pressure, a model able to dynamically return changes in volume of each of the three compartment and, as a sum, of the entire chest wall [CW] has been developed [5]. The marker disposition for the three compartment chest wall model is shown in Figure 1. The number of used markers is 89, 42 placed on the front and 47 on the back of the subject.

**Figure 2.**
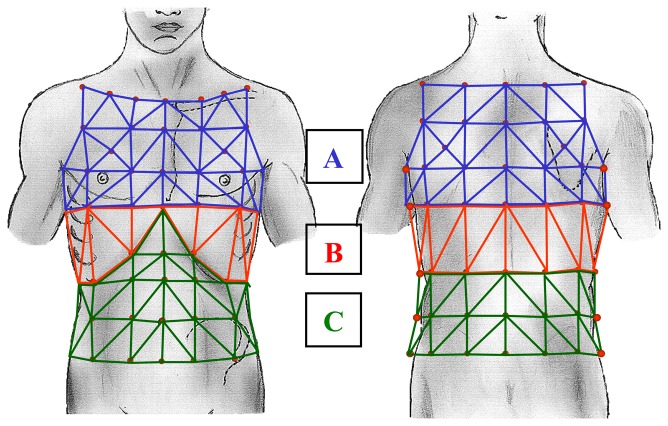
The three compartment chest wall model: A: Pulmonary apposed rib cage [RC,p]; B: abdominal apposed rib cage [RC,a]; C: abdomen [AB]; A+B+C = chest wall [CW].

To measure the volume of chest wall compartments from surface markers we define: 1) the boundaries RC,p as extending from the clavicles to a line extending transversely around the thorax at the level of the xiphoid process (corresponding to the top of the area of the apposition of the diaphragm to the rib cage at end expiratory lung volume in sitting posture, confirmed by percussion); 2) the boundaries of RC,a as extending from this line to the costal margin anteriorly down from the xiphosternum, and to the level of the lowest point of the lower costal margin posteriorly; and 3) the boundaries of AB as extending caudally from the lower rib cage to the level of the anterior superior iliac crest. The markers are placed circumferentially in seven horizontal rows between the clavicles and the anterior superior iliac spine. Along the horizontal rows the markers are arranged anteriorly and posteriorly in five vertical rows, and there was an additional bilateral row in the midaxillary line. The anatomical landmarks for the horizontal rows are: 1) the clavicular line; 2) the manubrio-sternal joint; 3) the nipples (∼ 5 ribs); 4) the xiphoid process; 5) the lower costal margin (10^th^ rib in the midaxillary line); 6) umbilicus; 7) anterior superior iliac spine. The landmarks for the vertical rows are: 1) the midlines; 2) both anterior and posterior axillary lines; 3) the midpoint of the interval between the midline and the anterior axillary line, and the midpoint of the interval between the midline and the posterior axillary line; 4) the midaxillary lines. An extra marker is added bilaterally at the midpoint between the xiphoid and the most lateral portion of the 10^th^ rib to provide better detail of the costal margin; two markers are added in the region overlying the lung-apposed rib cage and in the corresponding posterior position. This marker configuration has previously been validated in normal subjects, along with a sensitivity analysis which assesses accuracy in estimating change in lung volume as a function of marker number and position [5]. The solid representation of the tricompartimental model as described by the X-Y-Z co-ordinates of each marker is shown in Figure 2. When compared with the gold standard (water sealed spirometer) the accuracy in the volume change measurements of the 89 markers model is very high, showing volume differences smaller than 5% [5].

## 3. Applications

### 3.1. Physiology

#### 3.1.1. Basic Physiologic Approach to Chest Wall Dynamics during Exercise

The possibility of combining the compartmental volume changes with the pressure acting on the same compartment is a powerful method of analysis of the behaviour of thoraco-abdominal muscular groups. The simultaneous acquisition of kinematic signals with pleural and gastric pressures during a relaxation manoeuvre allows the representation of pressure-volume plots describing the mechanical characteristics of each chest wall compartment. The plots of dynamic pressure-volume loops, obtained in different conditions, over imposed on the relaxation line let the pressure developed by different muscle groups to be measured. Two studies concerning chest-wall mechanics during either cycling or walking exercise in normal humans [7, 9] have shown that abdominal muscles are maximally contracted at the end of expiration, gradually relaxed throughout the inspiration (abdominal volume to gastric pressure dynamics loops had no hysteresis), and fully relaxed only at the end of inspiration. This behaviour resulted in a progressive fall in abdominal pressure throughout inspiration, even at warm up of maximal work load, in contrast with the normal rise in abdominal pressure during resting breathing. This mechanism that minimises transdiaphragmatic pressure let the diaphragm contract quasi-isotonically. So in this condition the diaphragm seems to act as a flow generator rather than a pressure generator. In the same studies OEP was used to investigate another aspect of respiratory mechanics during exercise: the rib cage distortion, that is due to the different pressure acting on the volumes of lower (abdominal) and upper rib cage (non diaphragmatic inspiratory/expiratory muscles acting on volume of the upper rib cage, and diaphragm and abdominal muscles acting on volume of the lower rib cage). Rib cage volume distortion was measured on a plot of lower to the upper rib cage volumes by measuring the perpendicular distance of the distorted configuration away from the relaxation line, which represents the undistorted configuration, divided by the value of volume of the upper RC at the intersection point [6]. This result in a dimension-less number that when multiplied by 100 gives percent distortion. The volume distortion surprisingly was <1% [6]. Thus, during exercise, the diaphragm, rib cage and abdominal muscles are coordinated so that rib cage distortion, although measurable, is minimised. In particular, the progressive relaxation of abdominal muscles observed during inspiration could prevent volume of the lower rib cage from an unbalanced expansion with respect to volume of upper rib cage. Finally, the increase in velocity of shortening of the diaphragm more than the increase the change in transdiaphragmatic pressure accounts for the increase in the muscle power.

#### 3.1.2. Chest Wall and Respiratory Effort during Walking

Comparative data about the chest wall kinematics and respiratory muscle action in healthy subjects while walking at increasing gradient with a constant speed [AG], and at increasing speed with a constant gradient [AS] have never been done. With increasing gradient, subjects assume a different posture because they tend to lean forward moving the centre of gravity anteriorly; moreover, to stabilize, subjects tend to grip a support. In normal volunteers, leaning forward may result in a different use of respiratory muscles [10] and a greater ventilation capacity when their arms are braced than when they are unsupported at the side [11]. This effect may be related to the improved function of the muscles of the neck and shoulder girdle [12]. In evaluating the contribution of the respiratory muscles to respiratory effort sensation, how much of the resulting power a muscle generates is partitioned into pressure and how much into flow is a unique function the load the muscle acts against for a given neural drive. Therefore, respiratory effort sensation may relate to either pressure or velocity of shortening depending on the respiratory muscle load. On this basis Duranti *et al.* [13] hypothesized that walking at increased speed, or increasing gradient might have different effects on chest wall kinematics and respiratory muscle power components, and contribute differently to respiratory effort. The results of this study indicated that the general strategy adopted by the respiratory centres during different walking modes does not differ in terms of ventilation, chest wall kinematics and respiratory muscle power, but it does in terms of its partitioning into pressure and velocity of shortening. A combination of different patterns of flow and pressure generation made the respiratory effort sensation similar during AS and AG.

#### 3.1.3. Chest wall dynamics during externally imposed expiratory flow limitation

Having described the actions and control of respiratory muscles during normal exercise Aliverti *et al.* [14] determined how externally imposed expiratory flow limitation affected exercise ventilatory pump performance. They carried out a number of studies with different aims. Initially, they wished to determine the consequence of reduction in the velocity of expiratory muscle shortening. They hypothesized that imposed expiratory flow limitation by decreasing expiratory flow would have three major consequences: (i) it would increase expiratory muscle force measurable as an increase in expiratory pressure, (ii) it would decrease inspiratory time, and would increase inspiratory muscle velocity of shortening, (iii) this in turn would functionally weaken inspiratory muscles by decreasing their ability to generate force. They found that expiratory flow limited cycling exercise decreased the velocity of shortening of abdominal muscles which increased abdominal pressure [14]. This imposes a load overcome by pre-inspiratory diaphragmatic contraction. The increased velocity of shortening of both the diaphragm and rib cage inspiratory muscles gives rise to a reduction of their ability to generate pressure. Notwithstanding, diaphragmatic and rib cage muscle pressures increased indicating an increased central drive to all muscles secondary to hypercapnia, developed in all subjects.

The results suggest a vicious circle in which expiratory flow limitation decreases velocity of contraction of abdominal muscles, but increases abdominal pressure and exacerbates hypercapnia. The latter increases central drive, increasing abdominal pressure even more, and leading to further carbon dioxide [CO_2_] retention. In a companion paper the same researchers [15] tried to find out why and how expiratory flow limitation leads to difficult in breathing (rated using a modified Borg scale) with resulting impairment of exercise performance. Multiple stepwise linear regression revealed that the difference in expiratory muscle pressure between control incremental exercise to exhaustion and expiratory flow limited exercise accounted for about 70% of the variance in change in Borg, while change in diaphragmatic power added 12.5%. The study indicated that high expiratory pressures cause severe dyspnea and contribute to impair exercise performance. Moreover, breathing became similar to a Valsalva manoeuvre with a prolonged forced expiration interrupted by rapid inspirations that were to short for the reduced venous return to increase to normal levels. As there were very little information in the literature on the effects of Valsava's manoeuvre in continuous exercise Aliverti *et al.* [16] in a more recent study attempted to determine if imposition of expiratory flow limitation decreased systemic oxygen delivery by estimating cardiac output and measuring arterial oxygen saturation in exercising normal subjects. They tested the hypothesis that expiratory pressure and short duty cycle induced by expiratory flow limitation during exercise in normal subjects reduces cardiac output and impairs systemic energy supply. The set of data showed that a decrease in cardiac output due to a decrease in stroke volume by 10% with expiratory flow limitation, remained decreased for at least 90s. Concurrently arterial saturation decreased by 5%, abdominal pressure increased and duty cycle decreased by 43%. These data suggest that this combination of events lead to a decrease in venous return secondary to high expiratory pressure and a decrease in duty cycle which decreased oxygen delivery to working muscles by about 15%.

To estimate diaphragm fiber length from thoraco-abdominal configuration, Aliverti *et al.* [17] measured axial motion of the right-sided area of apposition by ultrasonography and volumes displaced by chest wall compartments (pulmonary, abdominal rib cage, and abdomen [Vab]) by OEP in four normal men during quiet breathing and incremental exercise without and with expiratory flow limitation. The axial motion of cephalic margin of the diaphragm in the area of apposition [D_ap_] was measured simultaneously with the volumes. Linear regression analysis between changes [Δ] in D_ap_ and the measured volume changes under all conditions showed that: (i) ΔD_ap_ was linearly related more to ΔVab than to changes in pulmonary and abdominal rib cage volumes; and (ii) this was highly repeatable between measures. Multiple stepwise regression analysis showed that ΔVab accounted for 89-96% of the variability of ΔD_ap_, whereas the rib cage compartments added <1%. The authors concluded that, under conditions of quiet breathing and exercise, with and without expiratory flow limitation, instantaneous axial motion of cephalic margin of the diaphragm (ΔD_ap_) can be estimated from ΔVab.

#### 3.1.4. Chest Wall Dynamics during Induced Hypercapnia

Studies on thoraco-abdominal motion in response to carbon dioxide [CO_2_] have shown that by decreasing end expiratory abdominal volume the expiratory muscles contribute to tidal volume [18, 19]. In contrast, it has been suggested by Henke *et al.* [20] using helium dilution that end expiratory lung volume does not change significantly during CO_2_ rebreathing. Other studies reported that a decrease in end expiratory lung volume is peculiar to exercise and should be associated with increased mechanical efficiency compared with CO_2_ inhalation. However, Romagnoli *et al.* argued that many of these results were questionable because a number of reasons (for details see ref. [21]). Thus the question still remained as to whether, regardless of the stimulus, the respiratory centers alter their output to the respiratory muscles in a manner appropriate to conserve coordinated activity. Based on substantial energetic cost when the chest wall compartments move independently [8] Romagnoli *et al.* [21] hypothesized that the increased motor output during CO_2_ is equally distributed to rib cage muscles and abdominal muscles such that the coordinated activity of respiratory muscles minimizes the pressure production of the diaphragm as it does during cycling [7] or walking [9]. To validate this hypothesis Romagnoli *et al.* [21] compared the response to CO_2_ rebreathing with those previously reported during walking [9] and cycling exercise [7].

During CO_2_ rebreathing test the progressive increase in end-inspiratory chest wall volume resulted from an increase in both end inspiratory volume of upper rib cage and volume of lower rib cage, while the progressive decrease in end expiratory chest wall volume was entirely due to the decrease in end expiratory abdominal volume. The end inspiratory increase and end expiratory decrease in volume of the chest wall were accounted for by inspiratory rib cage and abdominal muscle recruitment, respectively. The power of the diaphragm, rib cage muscles and abdominal muscles progressively increased. However, while most of diaphragm power was expressed in terms of velocity of shortening, most of power of rib cage and abdominal muscles was expressed as force or pressure. A comparison of CO_2_ results with data obtained during leg exercise revealed: (i) a gradual vs. an immediate response, (ii) a similar decrease in both abdominal volume and abdominal pressure, (iii) an apparent lack of any difference in abdomen recruitment, (iv) less gradual abdomen relaxation, (v) no drop in diaphragmatic pressure, but a similar diaphragm power change, and decrease in pressure-to-velocity ratio of the muscle.

In turn, the authors found that in healthy human the increased motor output with hypercapnia is equally distributed between rib cage muscles and abdominal muscles to minimize transdiaphragmatic pressure. Moreover, data on chest wall kinematics and respiratory muscle recruitment are only partly in line with those obtained during walking or cycling exercise.

#### 3.1.5. Chest Wall Kinematics and Mechanics during Arm Exercise. A Comparison with Leg Exercise

The coordinated respiratory muscle action translates into proportional changes in the volume of the CW compartments when human beings cycle, run or walk [7, 9, 13]. This complex interaction between the diaphragm, inspiratory rib cage muscles, and abdominal muscles is poorly understood during unsupported arm exercise [UAE]. Comparing UAE with leg exercise [LE] in normal subjects Celli *et al.* [22] found that UAE resulted in less ventilatory contribution of inspiratory muscles of the rib cage and more contribution by the diaphragm and abdominal muscles. In a two compartment rib cage model this shift in dynamic work results in rib cage distortion [6]. Romagnoli *et al.* [23] therefore hypothesized that a decrease in pressure contribution of the rib cage inspiratory muscles, and increase in pressure production of the diaphragm and abdominal muscles with UAE might be associated with rib cage distortion as opposed to undistorted configuration during LE at comparable ventilation. A major contribution of the rib cage inspiratory muscles to the respiratory effort sensation with a minor role of the diaphragm has been firmly established [24-26]. Recruitment of the expiratory muscles also increases the sensation of the respiratory difficulty in human beings [15, 22, 27]. Therefore, given the complexity of chest wall mechanics during UAE [22] Romagnoli *et al.* [23] hypothesized that different chest wall kinematics and muscle coordination may differently affect the sensation of respiratory effort during UAE and LE. The results showed that unlike LE, with UAE: (i) end inspiratory volume of the chest wall did not increase because of the lack of significant changes in end inspiratory volume of the pulmonary rib cage; a decrease in end inspiratory volume of the pulmonary rib cage contributed to a decrease in end expiratory volume of the chest wall, (ii) inspiratory pressure production of the rib cage muscles did not significantly increase from quiet breathing. No clear cut differences in rib cage distortion were found between UAE and LE. Finally, changes in abdominal muscle pressure predicted a consistent amount of variability in the Borg score (62%) with UAE, whereas change in pressure production of inspiratory rib cage muscles predicted ∼41% variability in the Borg score with LE. What is the clinical relevance of this study? Based on the present results and those in patients with ankylosing spondylitis and rib cage rigidity [28] we speculate that diverting rib cage muscles from ventilatory function to postural function limits inspiratory rib cage expansion more than some degree of rib cage rigidity does. This may have negative ventilatory consequences in severely hyperinflated patients with chronic obstructive pulmonary disease [COPD] who unlike the subjects of the present study are not able to deflate the rib cage and abdominal compartments to maintain an adequate tidal volume when using rib cage muscles for daily living activities.

#### 3.1.6. Phonetic Tasks (Speech Activities)

Previous studies have mainly focused on speech breathing [29, 30]. Phonetic tasks impose respiratory demands in terms of both expiratory flows and expiratory excursions in lung volume [30, 31]. With speech, end expiratory lung volume decreases below functional residual capacity and essentially impinges on maximal expiratory flow volume curve in normal subjects [30]. These findings point out the importance of large expiratory reserve volume and flow reserve for phonation. The use of large excursion below functional residual capacity indicates that a primary abdominal strategy is used to produce sounds rather than increase expiratory elastic recoil pressure [29, 30]. Binazzi *et al.* [32] firstly systematically studied breathing phonation in a group on normal young men and a group of young women. Binazzi *et al.* [32] used OEP to assess breathing movements and to provide a quantitative description of chest wall kinematics during phonatory tasks: reading aloud and singing at comfortable effort and during high effort whispering which requires greater flows and volume excursions than normal reading or singing.

During phonation the breathing pattern was different from quiet breathing and exercise: (i) during phonation, tidal volume and expiratory time increased while inspiratory time decreased. The expiratory volume changes and flows during high effort whispering [HW] were considerably greater than during vocalization. During HW, the overall end expiratory thoracic volume significantly decreased as a result of decreased volume of all compartments, and essentially impinged on the maximal expiratory flow-volume curve, (ii) while, as previously shown, during exercise the expired volume is due entirely to the abdomen, during phonation all three chest wall compartments contribute to it. Under all conditions studied breathing was, on average, more costal in females than in males, but this was mainly related to different size rather than gender *per se*.

Physical characteristics have hence a greater importance than gender in determining breathing pattern and chest wall kinematics during phonation. The activity of the control of expiration during phonation is more complex than during exercise.

#### 3.1.7. Chest Wall Kinematics during Laughter and Playing

During laughter, stress is applied to the chest wall causing rapid and substantial motion [33-35]. The inability to measure chest wall compartmental volumes and motion without instruments that affect them prevented detailed descriptions of respiratory system dynamics. A detailed knowledge of the dynamic events occurring in the respiratory system during episodes of laughter allowed Filippelli *et al.* [36] to understand the pattern and magnitude of physiological reaction of the chest wall to such forceful mechanical events. They may be summarized as follows: (i) episodes were associated with a remarkably decrease in lung volume due to sudden and sustained increase in gastric and esophageal pressures. The latter substantially exceeded the critical pressure, thus generating expiratory flow limitation and dynamic collapse in the airway downstream from the choke point, (ii) higher transdiaphragmatic pressure at the end of the consecutive expiratory efforts that during a slow expiratory manoeuvre over the same absolute lung volumes suggested that the diaphragm tended to counteract the excess the pressure generated on the abdominal site thus protecting intrathoracic structures from further mechanical stress and compression. The data of the present study suggest that the exaggerated increase in pleural pressure that decreases functional residual capacity and causes expiratory flow limitation even for a few seconds might play a remarkable role in the pathogenesis of phenomena associated with laughter such as cataplexy, syncope episodes, bronchospasm.

Respiratory parameters and sound were recorded during professional flute playing in order to assess what physiological processes were associated with the control of sound production that results in “breath support” which in turn is associated with high quality playing. Four standing young professional flautists played flute excerpts with and without breath support. Recordings included OEP measurements of chest wall volume and its compartments, surface electromyography of the scalene, lateral abdominal, rectus abdominus, parasternal and sternocleidomastoid muscles, mouth pressure, and sound. Results showed that flute support entails antagonistic contraction of non-diaphragmatic inspiratory muscles that tends to hold the rib cage at higher lung volume. This relieves the expiratory muscles from the task of producing the right mouth pressure, especially at the end of the phrases, so they can contribute more to the finer control of mouth pressure modulations required for high quality playing [37].

#### 3.1.8. Chest Wall Kinematics during Coughing

The study of kinematics of the chest wall allowed us to define the relative deflationary contribution of its compartments during fits of coughing. The three-compartment model of the chest wall dictates that contraction of the abdominal muscles has both a deflationary action on the lower rib cage via their insertional components (the rectus and obliquus muscles), and an inflationary one via their non-insertional components (the trasversus muscle), the net effect being such that the upper rib cage deflation is commensurate with lower rib cage deflation [6]. However, if forces applied to the upper rib cage are out of proportion with those applied to lower rib cage, distortion might ensue during fits of coughing. In this way the abdominal rib cage is exposed to greater positive abdominal pressure at the end of expiration during cough [38]. Lanini *et al.* [39] therefore hypothesized that uneven distribution of operating forces may results in rib cage distortion during coughing. The results obtained in 12 (five women) healthy subjects during voluntary single and prolonged coughing efforts at functional residual capacity and after maximal inspiration (max) showed that the tree chest wall compartments contributed to reducing end expiratory volumes of the chest wall during cough at functional residual capacity and prolonged maximum cough, with the latter resulting in the greatest chest wall deflation. Mean rib cage distortion, calculated by plotting the loop of the volume of upper rib cage to that of lower rib cage (see also chapter I, Technical procedure) did not differ between men and women, but tended to significantly increase from single to prolonged cough max. Lanini *et al.* [39] therefore concluded that rib cage distortion may ensue during coughing, probably as a result of uneven distribution of forces applied to the rib cage. Moreover, in confirming unpublished data by Morris *et al.* [40] we found that the chest wall did not significantly differ between men and women during fits of coughing.

### 3.2. Clinical Applications

#### 3.2.1. Asthma

Gorini *et al.* [41] assessed the chest wall mechanics during histamine-induced bronchoconstriction in asthmatic patients. The aims were the following: first, to assess the degree of hyperinflation associated with acute bronchoconstriction and its partitioning into chest wall compartments; second, to assess the relationships between hyperinflation and respiratory muscle recruitment and interaction.

At maximum level of bronchoconstriction (FEV_1_ 57.2% of the control value) and total lung resistances (RL: 15.8 [3.5] cm H_2_O/L/s) all patients but one were hyperinflated, with the mean increase in end expiratory volume of the chest wall being 0.72 L (23.6% of control functional residual capacity). The volumes of rib cage compartments accounted for 89.9% of the volume of hyperinflation, whereas abdominal volume accounted for the remaining 10.1%. The plots of volumes of upper to lower rib cage were close to their relaxation line demonstrating that the hyperinflation volume were shared proportionally by both the rib cage compartments. The analysis of the volume-pressure diagram plotted on their relaxation curves showed that dynamic loops of chest wall volume vs pleural pressure, and volume of the upper rib cage vs pleural pressure were totally displaced leftward the relaxation line such that the maximum (less negative) expiratory pleural pressure during breathing was less than relaxation pressures of chest wall and pulmonary-apposed rib cage. This provided evidence that hyperinflation was associated with persistent activity of the rib cage inspiratory muscles throughout expiration. The analysis of the plot abdominal volume vs gastric pressure indicated that, during increasing bronchoconstriction, abdominal muscles were recruited progressively as indicated by the rightward shifting of dynamic loops. These data indicate that in asthmatic patients: (i) the rib cage largely accounts for the volume of hyperinflation associated with acute bronchoconstriction, whereas phasic abdominal muscle recruitment during expiration minimizes the increase in end expiratory volume of the abdomen in the majority of patients, (ii) pulmonary-apposed rib cage and diaphragm-apposed rib cage contribute proportionally to the hyperinflation such that volume distortion of the rib cage at end expiration was negligible.

Dynamic hyperinflation during acute bronchoconstriction is an important determinant of dyspnea in patients with asthma [42] in whom the indirect method for assessing dynamic hyperinflation based on measure of inspiratory capacity is questionable because with bronchoconstriction the total lung capacity may be reduced. A useful alternative is provided by OEP which estimates both the overall volume of the chest wall and that of its components, [5, 9, 36, 41]. The increased volume of the rib cage and abdomen may contribute to dyspnea during methacholine induced airway narrowing. Filippelli *et al.* [43] have shown that dynamic hyperinflation, that is, the increase in end expiratory volume of the chest wall was due to increase in volume of the rib cage and to a lesser extent abdominal volume. Multiple regression analysis with dyspnea (by a modified Borg scale) as a dependent variable showed that changes in end expiratory volume of the CW and an index of airway obstruction (FEV_1_) explained 56% of variance in Borg score, with the former explaining 48% of it. These data support the contention that two mechanisms dynamic hyperinflation and dynamic compression of the airway contribute to dyspnea.

#### 3.2.2. Chronic Obstructive Pulmonary Disease (COPD)

##### 3.2.2.1. Methods

Direct measurements of resting functional residual capacity need sophisticated techniques that are not always available in every pulmonary function testing laboratory. In contrast inspiratory capacity, i.e., the difference between total lung capacity and functional residual capacity can be easily measured even with a simple spirometer and may accurately track the changes in functional residual capacity after bronchodilation if total lung capacity remains constant [44]. Thus inspiratory capacity has been proposed for inclusion in the criteria for reversibility of airway obstruction [44-47]. Nevertheless, accurate physiologic validation of inspiratory capacity for this use has never been done probably due to the technical difficulties to prove that total lung capacity is stable with the bronchodilator agents. The versatility to measure lung volumes under a variety of functional conditions prompted Duranti *et al.* [48] to turn this techniques with the specific purpose to validate inspiratory capacity for the bronchodilator test in patients with COPD. The result of the study showed a significant increase in volume of the chest wall at functional residual capacity, but not change in volume of the chest wall at total lung capacity. Inspiratory capacity measured with pneumotachograph correlated with, and was not significantly different from inspiratory capacity measured with OEP. Thus the data validated the use of inspiratory capacity to measure changes in functional residual capacity in the assessment of reversibility of airway obstruction [48].

##### 3.2.2.2. Clinics

Pursed lip breathing [PLB] is a breathing retraining strategy often spontaneously employed by patients with COPD to relieve dyspnea [49-52]. However, despite improvement in gas exchange [50, 53] and efficiency of ventilation the efficacy of PLB in relieving dyspnea varies greatly among patients [51, 54-56]. In contrast to dynamic hyperinflation, reduction in lung hyperinflation relieves dyspnea [44, 57]. Based on varying changes in end expiratory lung volume with PLB [52, 54] Bianchi *et al.* [58] hypothesized that the effect of PLB on breathlessness relies on its deflationary effects on the chest wall. They found that compared with spontaneous breathing, patients exhibited significant reduction in end expiratory volume of the chest wall and a significant increase in end inspiratory volume of the chest wall. The former was mostly due to the decrease in end expiratory volume of the abdomen related to increase in expiratory time, but not to baseline functional residual capacity. In a stepwise multiple regression analysis decrease in end expiratory volume of the chest wall accounted for 27% of the variability in Borg score. The data indicate that lengthening expiratory time PLB deflates the lung and reduces dyspnea. In this connection more recently, Bianchi *et al.* [59] identified the reason why some patients benefit from PLB. The significant difference in increased in tidal volume they found in two groups of patients translates into a different PLB effect on dyspnea. The ability of PLB to expand chest wall tidal volume with the expiratory abdominal contribution limits the inspiratory work of breathing thus modulating the sensation of dyspnea.

Last, but not the least, Binazzi *et al.* [60] have recently contributed to the assessment of the pathophysiology of the so-called Hoover's sign. Clinical evidence of the diaphragm's vulnerability in the effect of hyperinflation is abundant in patients with COPD [61]. One indicator of diaphragm dysfunction is the Hoover's sign [62], consisting of an inward movement of the lower lateral rib cage during inspiration. However, the basis of abnormal rib cage motion and the effect of hyperinflation of rib cage have not been systematically examined in these patients. Some factors argue against a close relationship between Hoover's sign and hyperinflation [63-65]. Binazzi *et al.* [60] therefore asked whether hyperinflation would produce rib cage distortion per se and hypothesized that lung hyperinflation and rib cage distortion (Hoover's sign) could independently define the functional condition of COPD patients. Binazzi *et al.* [60] based the hypothesis on the following observation: (i) a remarkably direct correlation has been found between abdominal rib cage compliance and distortability [66], and (ii) passive tension in the abdominal muscle exerts an important deflationary action on abdominal ribs during inspiration [6]. We found that COPD patients may accommodate less volume in upper rib cage and, as a corollary, more volume in the abdominal compartment that normal subjects. Volumes of upper rib cage, lower rib cage and abdomen, and the percentage of absolute volume of the chest wall. Chest wall, upper rib cage, lower rib cage, their ratio, and volume of the abdomen quantified Hoover's sign, but did not correlate with the level of hyperinflation. In agreement with previous findings [67,68] the observation of a lack of any significant relationship between quantitative indices of Hoover's sign and functional residual capacity validates the starting hypothesis that rib volume distortion cannot be fully explained by static hyperinflation in these patients. Thus OEP quantitative method helps to measure the presence and degree of Hoover's sign and to evaluate the agreement between clinical and quantitative analysis. Rib cage distortion and hyperinflation appear define independently the functional condition of these patients.

##### 3.2.2.3. Physical Activities

Distinct sensations of dyspnea during exercise [69] are associated with dynamic hyperinflation and its negative mechanical effects: threshold load and the uncoupling of the normal association between inspiratory effort and ventilatory output [44, 69, 70]. A decrease in inspiratory capacity, a mirror of increase in dynamic hyperinflation, along with a change in tidal volume and respiratory frequency account for 61% of the variance in rating of breathing difficulty in exercising patients with COPD [44]. Even though hyperinflation maximizes tidal expiratory flow rates breathing at high lung volumes has serious mechanical and sensory consequences: (i) tidal volume becomes positioned closer to total lung capacity where there is a significant elastic loading to the inspiratory muscles [44], (ii) shortening of the operating length of the inspiratory muscles compromises their ability to generate pressure, (iii) inspiratory muscles are forced to use a large fraction of their maximal force-generating capacity during tidal volume [44, 70, 71], (iv) effort production without an adequate concurrent volume or flow reflects the neuro-ventilatory dissociation of the respiratory pump [44]. Although there is much evidence of dynamic hyperinflation during exercise in many cases, several more recent studies also indicate that this is not always the case in patients with COPD [72]. This is in keeping with earlier data suggesting that patients with COPD could also be limited by fatigue of their leg muscles when they exercise rather than simply by ventilatory factors [73-75]. Furthermore, Aliverti and Macklem [76] and Aliverti *et al.* [77], by measuring the total chest wall volume non-invasively using OEP, have recently shown that exercise limitation and its attendant dyspnea are not necessarily associated with dynamic pulmonary hyperinflation in COPD. Unlike patients who hyperinflated, a significant number of patients (*euvolumics*) reduced abdominal volume, thus preventing dynamic hyperinflation. The most likely explanation was a difference between groups in their resting tidal expiratory flow limitation associated with hyperinflation. Moreover, despite their better flow reserve, *euvolumics* reduced end expiratory-thoracic-volume largely by reductioning the volume of their abdominal compartment. To do that, *euvolumics* developed high intra-abdominal pressure rather than permitting end expiratory-thoracic-volume to rise as in patients who hyperinflate (*hyperinflators*). It is worth noting that in the study [77] the pattern of responses (*euvolumics vs hyperinflators*) was associated with similar intensity of breathlessness.

#### 3.2.3. Pathology of the Rib Cage

Recent evidence indicates that abdominal muscles are important contributors to ventilation in healthy humans [7, 9, 19, 21, 78]. The gradual inspiratory relaxation of the abdominal muscle during hyperventilation helps substantially increase the volume of the chest wall and lets the diaphragm to act as a flow generator [7, 9, 21]. Therefore, Romagnoli *et al.* [28] hypothesized that in patients with Ankylosing spondylitis [AS], a pathological process which involves fusion of costovertebral and sternoclavicular joints along with intercostal muscle atrophy and limited rib cage expansion, a respiratory center strategy to help the diaphragm function may involve the coordinated action of the diaphragm and abdominal muscles. To validate this hypothesis in six AS patients Romagnoli *et al.* [28] assessed the ventilatory response to carbon dioxide [CO_2_] by combined analysis of CW kinematics and respiratory muscle pressure. During CO_2_ rebreathing chest wall expansion increased to a similar extent in patients to that in controls: however, the abdominal component increased more and rib cage component less in patients. Peak inspiratory rib cage muscle pressure, but not abdominal muscle pressure, was significantly less in patients than in controls. End inspiratory transdiaphragmatic pressure increased similarly in both groups whereas inspiratory swing in transdiaphragmatic pressure increased significantly only in patients. No pressure, or volume signals correlated with disease severity. Thus the diaphragm and abdominal muscles help expand the chest wall in AS patients regardless of the severity of their disease. These findings support the starting hypothesis that a coordinated response of respiratory muscle activity optimises the efficiency of the thoracoabdominal compartment in conditions of limited rib cage expansion. An understanding of the chest wall dynamics is essential to individual tailoring of rehabilitation programmes for AS patients.

#### 3.2.4. Neuromuscolar Disease

##### 3.2.4.1. Chest Wall Kinematics in Patients with Hemiplegia

Focal destructive hemispheric lesions result in controlateral dysfunction of ventilatory muscles [79-81]. Employing electromyography [EMG] [79] and ultrasonography [80], more recent studies have clearly shown a reduction in EMG activity and movement on the paretic side during voluntary ventilation. However, in hemiplegia, results with volitional hyperventilation differ from those with chemical stimuli. Klassen and coworkers [82] showed, compared with control subjects, a significantly higher carbon dioxide [CO_2_] sensitivity, apparently due to the increase in both respiratory frequency and tidal volume. The authors hypothesized that the increase in CO_2_ sensitivity was related to a loss of normal inhibitory or “damping” influences on the brain stem–mediated ventilatory response to hypercapnia. However, owing to technical difficulties, no evidence of asymmetrical ventilation of the two sides of the chest wall has yet been provided with hypercapnia; so it is still unknown whether volitional ventilation and the ventilatory response to CO_2_ are asymmetrical. Lanini *et al.* [83] reasoned that if the inhibitory control of the cortex is unilateral and each cerebral hemisphere inhibits the ventilatory response to CO_2_ of the opposite side of the chest wall, inhibition of the CO_2_ ventilatory response would be less on the paretic side in patients with a unilateral hemispheric stroke, thereby resulting in a greater ventilatory response than on the healthy side. On the other hand, during voluntary hyperventilation [VH], the ventilatory response would be lower on the paretic than on the healthy side. Lanini *et al.* studied eight patients with hemiparesis and nine normal sex- and age-matched subjects. Right- and left-sided tidal volume [VT] was reconstructed using OEP. In control subjects, no asymmetry was found in the study conditions. VTs of paretic and healthy sides were similar during quiet breathing, but paretic VT was lower during voluntary hyperventilation in six patients and higher during hypercapnic stimulation in eight patients (p = 0.02). The ventilatory response to hypercapnic stimulation was higher on the paretic than on the healthy side (p = 0.012). The data validate the starting hypothesis that hemiparetic stroke produces asymmetric ventilation with an increase in carbon dioxide sensitivity and a decrease in voluntary ventilation on the paretic side.

##### 3.2.4.2. Duchenne Muscular Dystrophy [DMD]

Aliverti *et al.* [84] studied DMD patients who were significantly less able than controls to expand their upper thoracic segments and increase their lung volumes. The kinematic capacity and ability of patients to increase volumes in the abdominal and upper thoracic regions by deep breathing decreased with the severity of disease. Among the patients with pulmonary restriction and inspiratory muscle strength at an intermediate level those with higher kinematic reserve maintained a significantly more normal ventilatory capacity and more normal nocturnal oxygen saturation than those with a lower kinematic reserve. Aliverti *et al.* [84] concluded that kinematic analysis can be helpful in determining differences in regional lung mobility and that a decrease in kinematic reserve correlates with the risk of nocturnal ventilatory dysfunction in DMD patients. Therapeutic intervention must be addressed at maintaining thoraco-abdominal kinematic reserve and lung compliance in such patients.

##### 3.2.4.3. Cough ineffectiveness

Neuromuscular diseases are characterized by progressive loss of muscle strength resulting in cough ineffectiveness with deleterious effects on the respiratory system [85, 86]. Assessment of cough effectiveness is therefore a prominent component of the clinical evaluation and respiratory care in these patients. Owing to uneven distribution of muscle weakness in neuromuscular patients [87] Lanini *et al.* [88] hypothesized that forces acting on the chest wall may impact of the compartmental distribution of gas volume resulting in a decrease in cough effectiveness. The current authors have shown that unlike controls patients were unable to reduce end expiratory chest wall volume and exhibited greater rib cage distortion during cough. Peak cough flow was negatively correlated with rib cage distortion, the greater the former the smaller the latter, but not with respiratory muscle strength. Therefore, insufficient deflation of chest wall compartments and marked rib cage distortion resulted in cough ineffectiveness in these neuromuscular set of patients.

#### 3.2.5. OEP in Intensive Care Unit

Chest wall volumes depend on the regional mechanical characteristics of the lungs and chest wall. Patients with acute lung injury [ALI] and acute respiratory distress syndrome [ARDS] typically have unevenly distributed lesions throughout the lung parenchyma and altered mechanical properties of the chest wall, especially after abdominal disease [89]. Aliverti *et al.* [90] used OEP to study eleven normal subjects during quiet and deep breathing, six sedated and paralyzed patients with acute lung injury and acute respiratory distress syndrome [ALI/ARDS] receiving continuous positive pressure ventilation [CPPV] [positive end-expiratory pressure [PEEP]=10 cm H_2_O, tidal volume = 300, 600, 900 ml], and seven ALI/ARDS patients receiving pressure support ventilation [PSV] (PEEP 10 cm H_2_O, pressure support = 5, 10, 15, 25 cm H_2_O). The volumes measured using OEP were compared with measurements taken using spirometry and pneumotachography. The three methods were highly correlated. Abdominal contribution to inspired volume was greater for normal subjects than for PSV patients (63 ± 11% versus 43 ± 14%, p < 0.001). It decreased with tidal volume for normal subjects (48.5 ± 15%, p < 0.05), whereas it increased for CPPV patients (61 ± 10%, p < 0.05). No significant distribution differences were found between 5 and 25 cm H_2_O PSV. The authors concluded that OEP is a feasible technique able to provide unique data on the distribution of chest wall volume changes in intensive care patients.

During PSV a part of the breathing pattern is controlled by the patient, and synchronization of respiratory muscle action and the resulting chest wall kinematics is a valid indicator of the patient's adaptation to the ventilator. The aim of the study of Aliverti *et al.* [91] was to analyse the effects of different PSV settings on ventilatory pattern, total and compartmental chest wall kinematics and dynamics, muscle pressures and work of breathing in patients with acute lung injury. In this study, conducted in a group of mechanically ventilated non-COPD patients with severe-to-moderate respiratory failure, Aliverti *et al.* [91] found that respiratory rate and tidal volume changes were good bedside indicators of work of breathing and respiratory drive. Furthermore, pressure support levels below 15 cm H_2_O increased the following: (i) the pressure developed by the inspiratory muscles, and the contribution of rib cage compartment to the total tidal volume, (ii) the simultaneous post-inspiratory action of the rib cage muscles, and expiratory action of the abdominal muscles, and (iii) the phase shift between rib cage and abdominal compartments.

## 4. Conclusions

We have described the technical limitations which affect the computation of thoraco-abdominal volume displacement and the characteristics that an ideal system should have. The OEP system is an optoelectronic device able to track the three-dimensional co-ordinates of a number of reflecting markers placed non-invasively on the skin of the subject. The simultaneous acquisition of kinematic signals with pleural and gastric pressures during a relaxation manoeuvre allows the representation of pressure-volume plots describing the mechanical characteristics of each compartment. The results of studies concerning chest wall dynamics by applying OEP system in a number of physiological and clinical experimental conditions are described.
